# Replantations

**Published:** 1879-01

**Authors:** J. C. Henry


### REPLANTATIONS.

BY J. C. HENRY, M. D.

Having had very general success in this practice for several months, I suggest my practice to others with the hope of similar results. Two assertions, First, it is the only successful way of curing an abscess, second, for exposed pulps replantation is certainly the most satisfactory.

Aside from the danger of breaking the tooth in extracting, or destroying the alveolar process, you can promise the patient a successful operation.

Fill the pulp cavity with a gold wire, it is the speediest and most complete. If filled with gold foil or amalgam there is danger of a portion becoming detached and causing alveolar abscess.

For a time I was discouraged, because of great pain in inserting the tooth. This I concluded was caused by the pressure upon the severed nerve. I now cut a groove in the root from the apex to the neck—and being sure there is no coagulated blood in the socket—insert the tooth with scarcely any pain. In the lower jaw gravitation will keep them in place. If there is no direct pressure upon the upper by the lower teeth, then a plan must be devised by which they can be retained until union takes place. For this purpose I use rubber dam and ligatures.

